# Purification, Structural Characterization, and Antitumor Activity of a Polysaccharide from *Perilla* Seeds

**DOI:** 10.3390/ijms242115904

**Published:** 2023-11-02

**Authors:** Hui Li, Ming Liu, Zikun Liu, Li Cheng, Mengsha Li, Chongwei Li

**Affiliations:** 1 Engineering Research Center of Agricultural Microbiology Technology, Ministry of Education, Heilongjiang University, Harbin 150500, China; 2221646@s.hlju.edu.cn (H.L.); mingliu9595@163.com (M.L.); liuzikun98@163.com (Z.L.); chengli@hlju.edu.cn (L.C.); 2 Heilongjiang Provincial Key Laboratory of Ecological Restoration and Resource Utilization for Cold Region, School of Life Sciences, Heilongjiang University, Harbin 150080, China; 3 Institute of Nature and Ecology, Heilongjiang Academy of Sciences, Harbin 150080, China

**Keywords:** *Perilla* seed, polysaccharide, antitumor activity, structural characterization

## Abstract

A previous study found that a crude *Perilla* seed polysaccharide (PFSP) fraction exhibited obviously antitumor activity; however, the structural characterization and antitumor properties of this polysaccharide remain unclear. In this study, the PFSP was extracted and purified via combined column chromatography, and the structure of a single polysaccharide fraction was characterized by methylation, IC, GC-MS, NMR, and AFM. The results demonstrated that the efficient antitumor polysaccharide fraction PFSP-2-1 was screened from PFSP with a relative molecular weight of 8.81 × 10^6^ Da. The primary structure of the PFSP main chain was →1)-Araf-(5→, →1,3)-Galp-(6→, →1)-Galp-(6→, →1,3)-Araf-(5→ and →1)-Xylp-(4→, and that of the side chains was →1)-Arap, →1,3)-Galp-(6→, →1)-Araf and →1)-Glcp-(4→, →1)-Galp-(3→ and →1)-Glcp, leading to a three-dimensional helical structure. CCK-8 experiments revealed that PFSP-2-1 significantly inhibited the growth of human hepatocellular carcinoma cells in vitro (*p* < 0.05), and its inhibitory effect positively correlation with the concentration of PFSP-2-1, and when the concentration of PFSP-2-1 was 1600 µg/mL, it showed the highest inhabitation rate on three hepatocellular carcinoma cells (HepG-2, Hep3b, and SK-Hep-1), for which the survival rates of HepG-2, Hep3b, and SK-Hep-1 were 53.34%, 70.33%, and 71.06%. This study clearly elucidated the structure and antitumor activity of PFSP-2-1, which lays a theoretical foundation for revealing the molecular mechanism of antitumor activity of *Perilla* seed polysaccharides and provides an important theoretical basis for the development of high-value *Perilla.*

## 1. Introduction

*Perilla frutescens* is an annual herb of the Labiatae family. It is the first medicinal and edible plant recognized by the Ministry of Health of China [1]. *Perilla* is undemanding and has good survival, and it is widely grown in Asian countries such as China, Korea, Japan, India, and Vietnam [2]. *Perilla* is rich in a variety of bioactive components, such as polysaccharides, flavonoids, phenolic acids, pigments, terpenoids, and fatty acids [3]. Some studies found that total flavonoids extracted from *Perilla* leaves can reduce blood lipid levels and lipid accumulation in adipose tissue, inhibit the formation of lipid peroxidation products, regulate the metabolic disorders of lipoproteins, increase the activity of antioxidant enzymes, and inhibit hyperlipidemia [4]. Moreover, anthocyanins from *Perilla frutescens* have been shown to induce apoptosis in Hela cells and inhibit tumor growth [5]. In addition, rosemarinic acid extracted from *Perilla* can inhibit epidermal inflammatory responses [6]. However, there are few studies of the structure and function of *Perilla* polysaccharides.

Polysaccharides are a kind of natural biological macromolecules formed by connecting 10 or more monosaccharides through glycosidic bonds; they exist in a wide range of animals, plants, algae, and microorganisms [7]. Plant polysaccharides demonstrated good biological activity and application value in terms of antitumor [8,9], antioxidation [10], hypolipidemic [11], hypoglycemic [12], and immunomodulation [13,14,15] activities. In terms of antitumor activity, plant polysaccharides have good biocompatibility and biodegradability and low toxicity, showing great application potential. The biological activities of plant polysaccharides are determined by the structure of the polysaccharide, including factors such as the molecular weight, monosaccharide composition, and type of glycosidic bond and higher structure [16]. It was found that five polysaccharide fractions with antitumor activity were isolated and purified from *ginger*, and structural characterization revealed that the five polysaccharides differed in terms of their type of glycosidic bond [17]. Small-molecule polysaccharides extracted from the root of *Platycodon grandiflorum* clearly inhibit the proliferation of lung cancer cell SPC-A-1 [18]. In addition, the inhibitory effect of *Lentinan* on sarcoma S-180 cells was found, and the inhibitory rate of *Lentinan* with a three-dimensional triple-helix structure was significantly higher than those of polysaccharides without a triple-helix structure [19]. Based on these findings, we can see that the structural composition of plant polysaccharides is complex, and the biological activities of different polysaccharide structural components are also significantly different. Therefore, the biological activities of different structures of *Perilla* polysaccharides may also change significantly. Unfortunately, so far, the structure of *Perilla* polysaccharides is not clear, and research into their anticancer activity has not been carried out. This creates a limitation to the development and utilization of *Perilla* polysaccharides.

In our previous study, crude polysaccharides of *Perilla* frutescens (PFSP) were extracted from *Perilla* frutescens seeds, and three main polysaccharide fractions (PFSP-1, PFSP-2, and PFSP-3) were obtained from *Perilla* frutescens after isolation and purification. Animal experiments demonstrated that the three polysaccharide fractions had a significant inhibitory effect on the growth of tumor cells in the H22-loaded mice, and the highest tumor inhibition rate was found in PFSP-2. In this study, PFSP-2 was further isolated and purified to obtain two fractions, PFSP-2-1 and PFSP-2-2, which were initially screened via in vitro antitumor experiments. Based on the viewpoint of inhibitory effect on hepatocellular carcinoma cells, PFSP-2-1 was more effective than PFSP-2-2. Therefore, this experiment was finalized to structurally characterize PFSP-2-1, and the structural composition and properties of this polysaccharide fraction were analyzed via methylation, IC, GC-MS, NMR, and AFM. This study provides a theoretical basis for the conformational relationship with the antitumor activity of *Perilla* polysaccharides and provides an important theoretical basis for the high-value development and application of *Perilla*.

## 2. Results and Discussion

### 2.1. Extraction and Purification of PFSP-2-1

**Figure 1:**
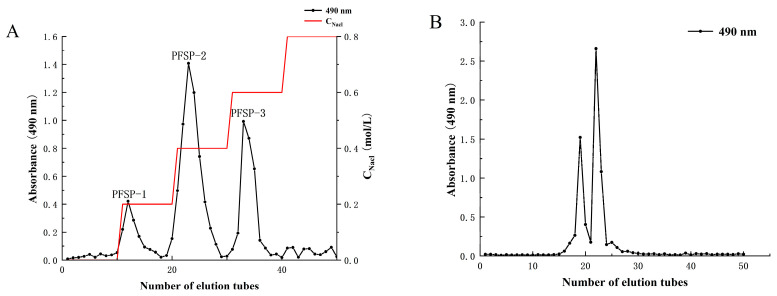
(**A**) Column elution profile obtained via ion-exchange chromatography using a DEAE-52 cellulose column; (**B**) column elution profile obtained using a Sephacryl S-500 HR gel filtration column.

The yield of crude polysaccharides from *Perilla* seed (PFSP) was 3.42% ± 1.97%. After separation in a DEAE-52 cellulose column (Figure 1A), three polysaccharide fractions were obtained, namely PFSP-1, PFSP-2, and PFSP-3. PFSP-2 had the highest polysaccharide content, with a yield of 11.25% ± 2.17%. PFSP-2 was further purified using a Sephacryl S-500 HR column (Figure 1B), and two elution peaks were obtained. The two components corresponding to the two peaks were collected separately, dialyzed, desalted, and freeze-dried. The calculated yields of the two components, namely PFSP-2-1 and PFSP-2-2, were 28.40% ± 2.36% and 36.3% ± 3.22%, respectively. In this study, we focused on PFSP-2-1 and analyzed its structure.

### 2.2. Characterization of PFSP-2-1

**Figure 2:**
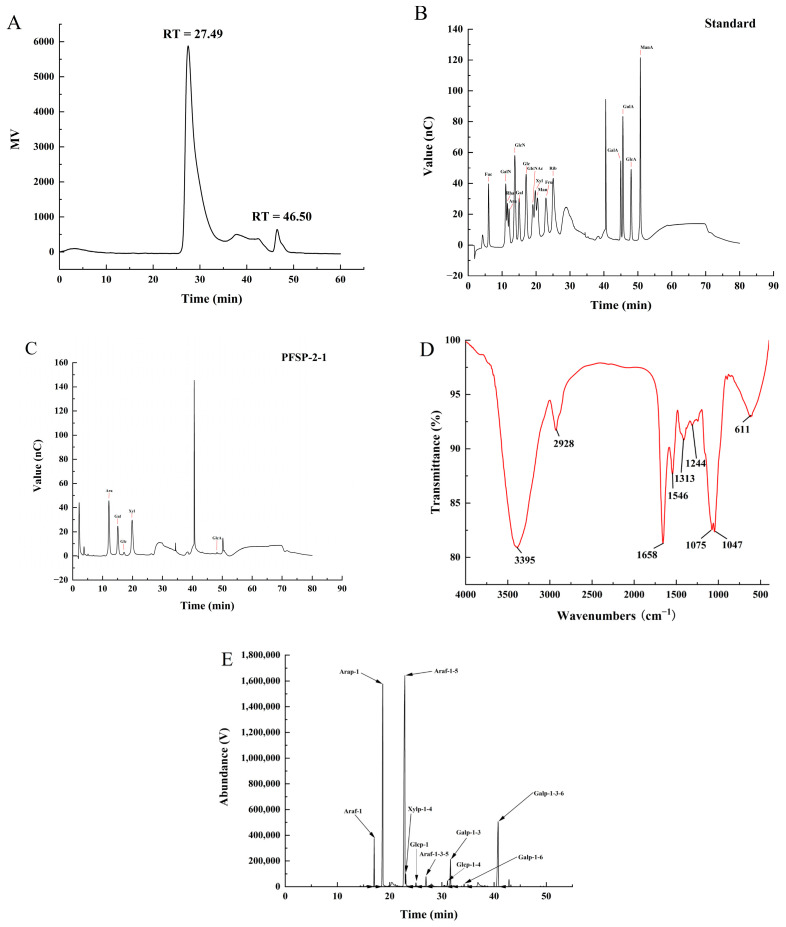
(**A**) The molecular weight and purity of PFSP-2-1; (**B**) standard chromatogram of mixed monosaccharides; (**C**) ion chromatogram of PFSP-2-1; (**D**) FT-IR spectrum; (**E**) GC–MS chromatogram of PFSP-2-1 (PMAA).

The structure of plant polysaccharides is complex, and studies have shown that their monosaccharide composition, molecular weight, type of glycosidic bond, and high-order structure are closely related to their biological activities. The purity and molecular weight of PFSP-2-1 were analyzed using HPGPC. A single peak was obtained in the HPGPC spectrum (Figure 2A), indicating that PFSP-2-1 was a homogeneous polysaccharide. The average molecular weight of PFSP-2-1 was calculated to be 8.81 × 10^6^ Da, primarily on the basis of a log Mw–RT calibration curve. The fitted curve of the glucuronide standard equation was *y* = 1.4186*x* + 0.47791, and the glucuronide content of PFSP-2-1 was found to be 16.22% ± 0.15% of the total. Qualitative and quantitative analyses of the monosaccharide composition of PFSP-2-1 via ion chromatography showed that PFSP-2-1 was mainly composed of arabinose, galactose, glucose, xylose, and glucuronic acid (Figure 2B,C), having a molar ratio of 20.207:11.223:1.228:18.232:0.331 in terms of the contents of each element.

The IR spectrum of PFSP-2-1 (Figure 2D) confirmed a wide and strong absorption peak at 3395 cm^−1^, caused by O–H stretching vibrations [20]. The absorption peak at 2928 cm^−1^ was associated with the bending vibration of the methyl C–H in the sugar ring. The absorption peak at 1658 cm^−1^ was assigned to the stretching vibration of COO^−^ [21], confirming the presence of glyoxalate. The weak absorption peak at 1546 cm^−1^ was attributed to –CO–NH–, indicating that PFSP-2-1 contained a small amount of protein [22]. The absorption band at 1244 cm^−1^ was considered to correspond to the C–H bending vibration [23]. The signal at 1313 cm^−1^ corresponded to the in-plane bending vibration of a saturated C–H. The two absorption peaks at 1075 cm^−1^ and 1047 cm^−1^ indicated that PFSP-2-1 contained pyranose rings [24].

The types of glycosidic bonds between the monosaccharide residues in PFSP-2-1 were investigated via methylation analysis (Figure 2E), and they were identified on the basis of the mass spectra of the ion fragments (Table 1). They mainly included →5)-Araf-(1→ (50.3%), Arap-(1→ (23.8%), and →3,6)-Galp-(1→ (13.2%). Based on the linkage structures of →3,5)-Araf-(1→ and →3,6)-Galp-(1, it was clear that PFSP-2-1 was a polysaccharide with a branched structure.

**Figure 3:**
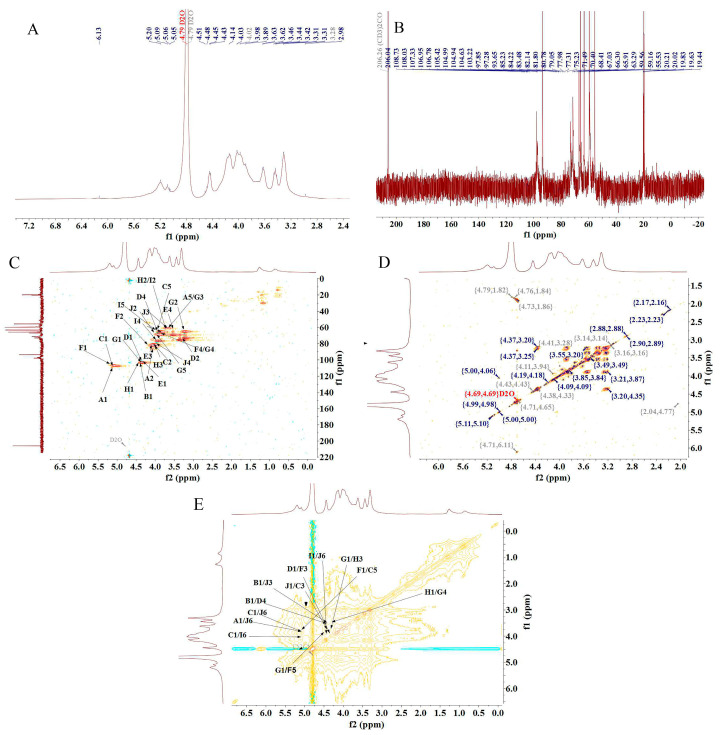
(**A**) ^1^H-NMR spectrum; (**B**) ^13^C-NMR spectrum; (**C**) HSQC NMR spectrum; (**D**) ^1^H–^1^H COSY NMR spectrum; (**E**) NOESY NMR spectrum.

The structure of PFSP-2-1 was further analyzed via NMR spectroscopy. In the ^1^H-NMR spectrum (Figure 3A), a massive number of proton resonance signals were concentrated in the δ 3.0–5.5 ppm region, with severe signal overlap. Multiple hetero-head proton coupling signals were discovered in the δ 4.5–5.5 ppm region [25]. In the ^13^C-NMR spectrum (Figure 3B), two hetero-head carbons were present in the δ 90–110 ppm region of PFSP-2-1, indicating that PFSP-2-1 contained both α- and β-glycosidic bonds. In particular, the signal at 108.73 ppm was characteristic of the arabinose end group, and that at 101.91 ppm corresponded to the xylose end group [26]. The presence of many signals in the δ 80–90 ppm range indicated the presence of furanose in PFSP-2-1. The chemical shift of the hetero-head carbon in furanose is often in the δ 105–110 ppm region, and the peak observed in this region was attributed to the hetero-head carbon of α-L-Araf [27].

There were 10 relatively significant hetero-head hydrogen peaks in the hydrogen region and hetero-head carbon peaks in the carbon region in the NMR spectrum. The chemical shift signals of the hetero-head region were found at δ 108.73 ppm, δ 105.42 ppm, δ 106.95 ppm, δ 101.91 ppm, δ 103.22 pm, δ 106.78, ppm, δ 104.94 ppm, δ 104.99 ppm, δ 104.63 ppm, and δ 103.22 ppm, sequentially named A, B, C, D, E, F, G, H, I, and J. After the attribution of heterodimeric signals, the results obtained via HSQC (Figure 3C), ^1^H–^1^H COSY (Figure 3D), and NOESY (Figure 3E) were combined with those from the ^1^H-NMR and ^13^C-NMR spectra, as well as the methylation results and the literature data. The ^1^H and ^13^C chemical shift signals of the main types of sugar residues in PFSP-2-1 were assigned based on the chemical shift data of similar sugar residue substitutions reported in the literature [28,29,30,31,32,33,34]. The results are reported in Table 2.

The NOESY spectrum provided information on intra- and inter-residue linkages totally on the basis of dipole correlation. In accordance with the NOESY spectrum (Figure 3E), a correlation peak was found between the heterodimeric proton signals of →5)-Araf-(1→ (δ 5.09) and H6 of →3,6)-Galp-(1→ (δ 3.85), as well as that of H6 of →6)-Galp-(1→ (δ 4.09), indicating the presence of →5)-Araf-(1→3,6)-Galp-(1→ and →5)-Araf-(1→6)-Galp-(1→ linkage fragments. A correlation peak between the heterodimeric proton signals of Arap-(1→ (δ 4.45) and H3 of →3,6)-Galp-(1→ (δ 3.63), as well as that of H4 of →4)-Xylp-(1→ (δ 3.83), indicated the presence of the linkage fragments of Arap-(1→3,6)-Galp-(1→ and Arap-(1→4)-Xylp-(1→. A correlation peak between the hetero-head proton signals of →3,6)-Galp-(1→ (δ 4.45) and its own H3 (δ 3.63), as well as the H5 of →5)-Araf-(1→ (δ 3.83), indicated the presence of →3,6)-Galp-(1→3,6)-Galp-(1→ and →3,6)-Galp-(1→5)-Araf-(1→. A correlation peak was observed between the heterodimeric proton signal of Araf-1→ (δ 5.09) and the H6 of →3,6)-Galp-(1→ (δ 3.85), indicating the presence of the linkage fragment of Araf-1→6,3)-Galp-(1→. The correlation peak between the heterodimeric proton signal of →4)-Xylp-(1→ (δ 4.40) and the H3 of →3,5)-Araf-(1→ (δ 4.04) indicated the presence of the linker fragment →4)-Xylp-(1→3,5)-Araf-(1→. The correlation peak between the heterodimeric proton signal of →4)-Glcp-(1→ (δ 4.44) and the H3 of →3)-Galp-(1→ (δ 3.98) indicated the presence of the linker fragment →4)-Glcp-(1→3)-Galp-(1→. The correlation peak between the heterodimeric proton signal of →3,5)-Araf-(1→ (δ 5.10) and the H5 of→5)-Araf-(1→ (δ 3.83) indicated the presence of the linker fragment →3,5)-Araf-(1→5)-Araf-(1→. The correlation peak between the hetero-head proton signal of →4)-Glcp-(1→ (δ 4.44) and the H5 of →3,5)-Araf-(1→ (δ 3.54) indicated the presence of the linker fragment →4)-Glcp-(1→5,3)-Araf-(1→. The correlation peak between the heterodimeric proton signal of →3)-Galp-(1→ (δ 4.46) and the H4 of →4)-Glcp-(1→ (δ 3.80) indicated the presence of the linker fragment →3)-Galp-(1→4)-Glcp-(1→. The correlation peak between the heterodimeric proton signal of →6)-Galp-(1→ (δ 4.46) and the H6 of →3,6)-Galp-(1→ (δ 3.85) indicated the presence of the linker fragment →6)-Galp-(1→6,3)-Galp-(1→.

**Figure 4:**
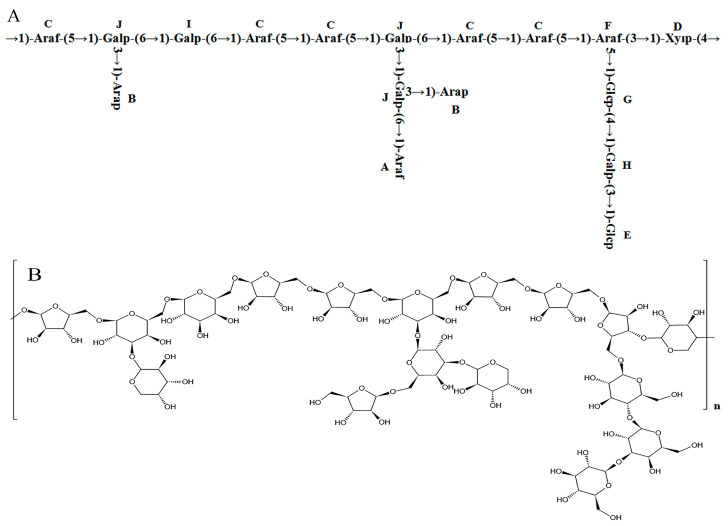
(**A**) Structure of PFSP-2-1 repeat unit; (**B**) diagram of the planar structure of PFSP-2-1.

Combining the ^1^H-NMR, ^13^C-NMR, COSY, HSQC, NOESY, and methylation analysis results, it was speculated that the main chain structure of PFSP-2-1 was →1)-Araf-(5→1)-Galp-(6→1)-Galp-(6→1)-Araf-(5→1)-Araf-(5→1)-Galp-(6→1)-Araf- (5→1)-Araf-(5→1)-Araf-(3→1)-Xylp-(4→, along with side-chain structures of →1,6)-Galp-(3→1)-Arap, →1,6)-Galp-(3→1,3)-Galp-(6→1)-Araf, →1,6)-Galp-(3→1)-Arap, and →1,3)-Araf-(5→1)-Glcp-(4→1)-Galp-(3→1)-Glcp. The major glycosidic bond of PFSP-2-1 consisted of →5)-Araf-(1→, Arap-(1→ and →3,6)-Galp-(1→, while the linkage structures of →3,5)-Araf-(1→ and →3,6)-Galp-(1 PFSP-2-1 indicated a polysaccharide with a branching structure [35]. We predicted the repetitive structural unit and planar structure of PFSP-2-1, and the results were shown in (Figure 4A,B).

**Figure 5:**
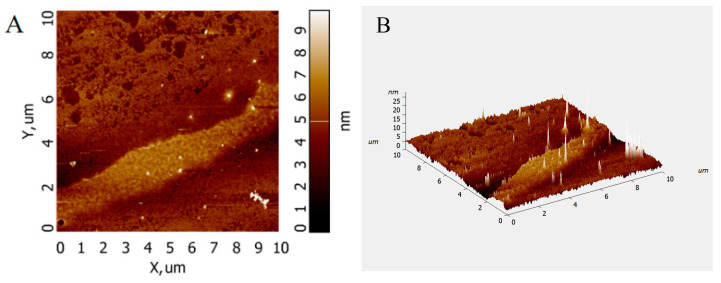
AFM scanning diagram of PFSP-2-1: (**A**) two-dimensional diagram; (**B**) three-dimensional diagram.

Atomic force microscopy (AFM) analysis allows the observation of the surface morphology of biomolecules and the conformation of glycan chains without sample pretreatment. The AFM images of PFSP-2-1 (Figure 5A,B) show that PFSP-2-1 presented an irregular spherical shape with a molecular diameter in the range of 0–25 nm, which is significantly larger than the average molecular diameters of individual polysaccharide chains (0.1–1.0 nm) [21], located at 15–50 nm in the polysaccharide triple-helix structure. This indicates that PFSP-2-1 had a branching structure, and the chains were intertwined with each other, probably forming a triple-helix structure.

**Figure 6:**
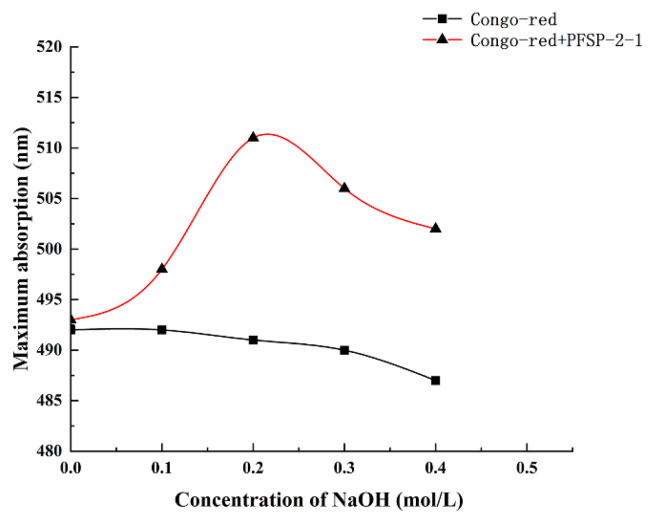
Different concentrations of NaOH affect the maximum absorption wavelength of the PFSP-2-1–Congo red complex.

The maximum absorption wavelengths of PFSP-2-1–Congo red complexes were determined in the presence of a NaOH solution at a concentration of 0–0.5 M (Figure 6). It was observed that with the increase in NaOH solution concentration, the maximum absorption wavelength of the control gradually decreased, whereas the maximum absorption wavelength of the polysaccharide–Congo red complex first increased and then decreased, reaching its highest value at the NaOH concentration of 0.2 mol/L. This indicates that under low-alkali conditions, the triple-helix structure of the polysaccharide formed a complex with the Congo red solution, whose maximum absorption wavelength was red-shifted compared to that of the control; however, when the NaOH concentration was increased beyond a certain value, the structure of the triple-helix was destroyed, as shown by the decrease in the maximum absorption wavelength. This indicates that PFSP-2-1 had a triple-helix spatial structure, consistent with the AFM results.

### 2.3. Antitumor Activity In Vitro

**Figure 7:**
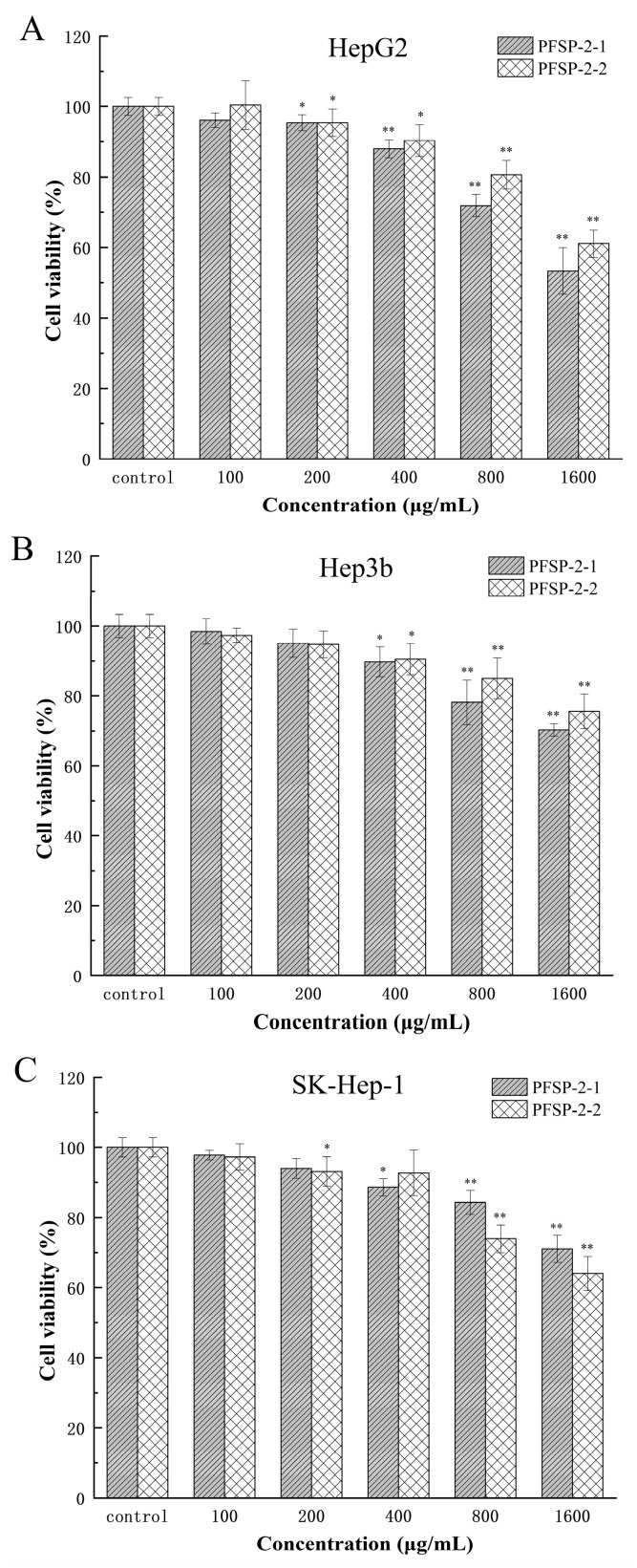
The effect of PFSP-2-1 on the viability of different cells: (**A**) HepG2 cells; (**B**) Hep3b cells; (**C**) SK-Hep-1 cells. The differences were statistically significant at * *p* < 0.05 and ** *p* < 0.01.

A CCK-8 experiments were used to determine the inhibitory effect of polysaccharide components on HepG2, Hep3b, and SK-Hep-1 cells, and the results are shown in Figure 7 and Table 3. Using the sample without polysaccharides as the control group, two kinds of polysaccharides (PFSP-2-1 and PFSP-2-2) had significant inhibitory effects on HepG2 cells (Figure 7A, *p* < 0.05). With the increase in polysaccharide concentration, the inhibition of PFSP-2-1 on HepG2 cells gradually increased. When the concentration was 100 µg/mL, PFSP-2-1 was not significant compared to CK (*p* > 0.05), and the cell survival rate was 96.12%. However, at the concentration of 200–1600 µg/mL, the inhibitory effect of PFSP-2-1 gradually increased, reaching its maximum effect at the a concentration of 1600 µg/mL, and the cell survival rate was 53.34%. The inhibitory effect of PFSP-2-2 was consistent with that of PFSP-2-1.

Compared to the control group, two kinds of polysaccharides (PFSP-2-1 and PFSP-2-2) had significant inhibitory effects on Hep3b cells (Figure 7B, *p* < 0.05). With the increase in polysaccharide concentration, the inhibition of PFSP-2-1 on Hep3b cells gradually increased. When the concentration was 100 µg/mL and 200 µg/mL, the effect of PFSP-2-1 was not significant compared to that of CK (*p* > 0.05), and the cell survival rates were 98.41% and 95.04%, respectively. However, at the concentration of 400–1600 µg/mL, the inhibitory effect of PFSP-2-1 gradually increased, reaching its maximum effect at a concentration of 1600 µg/mL, and the cell survival rate was 70.33%. The inhibitory effect of PFSP-2-2 was consistent with that of PFSP-2-1.

Compared to the control group, the two kinds of polysaccharides (PFSP-2-1 and PFSP-2-2) had significant inhibitory effects on SK-Hep-1 cells (Figure 7C, *p* < 0.05). Based on the inhibitory results of PFSP-2-1 on SK-Hep-1 cells, the inhibitory effects of 100 µg/mL and 200 µg/mL on SK-Hep-1 cells were not significant (*p* > 0.05), but the inhibitory effects of 400–1600 µg/mL on SK-Hep-1 cells was significant (*p* < 0.05). The inhibition results of PFSP-2-2 on SK-Hep-1 cells were different to those of PFSP-2-1, in which the inhibitory effect on SK-Hep-1 cells was not significant (*p* > 0.05) at the concentrations of 100 µg/mL and 400 µg/mL; however, at concentrations of 200 µg/mL, 800 µg/mL, and 1600 µg/mL, it had a significant inhibition effect (*p* < 0.05), reaching its maximum effect at a concentration of 1600 µg/mL, and the cell survival rate was 64.02%. In summary, the inhibitory effects of the polysaccharide fractions on different tumor cells were different. At the same concentration, the highest inhibition was observed in HepG2 cells, which are often used to screen for drugs, and the inhibitory effect of PFSP-2-1 was found to be more significant than that of PFSP-2-2 (*p* < 0.05).

### 2.4. Discussion

As natural biomacromolecules, polysaccharides’ structures are the key factors affecting their biological activity and physical and chemical properties. In this study, it was found that both polysaccharides (PFSP-2-1 and PFSP-2-2) had inhibitory effects on HepG2, Hep3b, and SK-Hep-1 cells, and the inhibitory effects gradually increased with the increase in the concentration (Figure 7). The monosaccharide composition of *Perilla* polysaccharide PFSP-2-1 is mostly arabinose and xylose, which is quite different from the previous report stating that glucose is the main monosaccharide composition in *Perilla* polysaccharides, which may be related to the variety of *Perilla* used and the methods for the extraction and purification the polysaccharides. At present, it has been found that hemicellulose in *Astragalus* residue mainly contains monosaccharides such as arabinose and xylose, which have an inhibitory effect on the proliferation of human lung adenocarcinoma cells and human normal lung epithelial cells [36]. In addition, arabinoxylan in wheat bran can significantly inhibit the growth of transplanted tumors in mice (*p* < 0.05) [37]. Therefore, it is speculated that arabinose and xylose in *Perilla* polysaccharides are some of the factors inhibiting the growth of liver cancer cells.

In addition to monosaccharide composition, molecular weight and glycosidic bond can be considered important structural features of polysaccharides that affect their biological activity. Studies have shown that polysaccharides with high molecular weights tend to have greater biological activity [38,39]. For example, researchers have studied the inhibitory effect of *Grifola frondosa* polysaccharides with different molecular weights on human lung cancer cells. The inhibitory rates of *Grifola frondosa* polysaccharides with high molecular weights on human lung cancer cells were significantly higher than those of other low-molecular-weight polysaccharides [40]. The reason for this may be that there are (1→3) α-D-Galp glycosidic bonds and a high content of (1→3,4) α-D-Galp in high-molecular-weight *Grifola frondosa* polysaccharides, and special glycosidic bonds are the key to the high antitumor activity of *Grifola frondosa* polysaccharides. We obtained high-molecular-weight *Perilla* PFSP-2-1 through separation and purification, and the glycosidic bonds were mainly →5)-Araf-(1→(50.3%) and Arap-(1→(23.8%). Previous studies found that the *Arachis* polysaccharide ALP-3 with the →5)-Araf-(1→, Araf-(1→, and →3,5)-Araf-(1→ branching structure was able to stimulate the immune activity of RAW264.7 cells better than the branched polysaccharide ALP-1 [34]. Therefore, it can be determined that the molecular weights and glycosidic bonds of *Perilla* polysaccharides have a certain influence on their antitumor activity.

Through NMR and methylation analysis, the main chain structure, side-chain structure, and glycosidic linkage mode of *Perilla* polysaccharide PFSP-2-1 were inferred, and it was determined that PFSP-2-1 was a polysaccharide with a branching structure. An AFM experiment and a Congo red experiment confirmed the triple-helix spatial structure of *Perilla* polysaccharides. It is generally believed that the higher structure of polysaccharides has a greater influence on the polysaccharides’ activity than the primary structure. At present, there are many studies of the triple-helix structure of *Lentinan*. For example, *Lentinan* with a triple-helix structure has a higher tumor inhibition rate in mice, and after being treated with DMSO, it becomes a single, flexible chain, which destroys its spatial configuration and greatly reduces its antitumor activity [41]. The antitumor activities of 10 kinds of *Lentinan* degraded via ultrasonic wave were investigated. Among them, the *Lentinan* with a single-stranded structure had no obvious antitumor activity, and the *Lentinan* with a triple-helix structure had the strongest antitumor activity. It can be seen that the advanced structure of polysaccharides has a greater influence on tumor activity than the primary structure [19]. At present, the research into the advanced structure of *Perilla* polysaccharides is insufficient, which limits the research into their antitumor activity.

## 3. Materials and Methods

### 3.1. Materials

*Perilla* meal was purchased from Heilongjiang Huainan Nongshengyuan Food Co., Ltd. (Jiamusi, China). HepG2, Hep3b, and SK-Hep-1 cells were donated by the Institute of Nature and Ecology, Heilongjiang Provincial Academy of Sciences. Phenol, sulfuric acid, chloroform, and n-butanol were purchased from Tianli Chemical Reagent Co., Ltd. (Tianjin, China). Petroleum ether, polyamide powder, anhydrous ethanol, acetic anhydride, perchloric acid, acetic acid, glacial acetic acid, methanol, sodium hydroxide, toluene, potassium bromide, and Congo red were purchased from Sinopharm Chemical Reagent Co., Ltd. (Shanghai, China). Trifluoroacetic acid, methyl iodide, sodium borohydride, ethyl acetate, dimethyl sulfoxide, and sodium hydride were purchased from Shanghai Adamas Reagent Co., Ltd. (Shanghai, China). DEAE-52 cellulose and Sephacryl S-500 HR propylene dextran gel columns were supplied by Beijing BoaoTuoDa Technology Co., Ltd. (Beijing, China). Monosaccharide standards (rhamnose, fucose, galactose, arabinose, xylose, glucose, mannose, ribose, fructose, glucuronic acid, galacturonic acid, amino galactose hydrochloride, N-acetyl-glucosamine, glucosamine hydrochloride, mannuronic acid, and gulo-glucuronic acid) and methylation kits were supplied by Borealis Biotechnology (Yangzhou, China). Sigma (St. Louis, MO, USA) supplied the dextran standards. High-sugar DMEM, MEM, and RPMI-1640 media were supplied by Solarbio (Beijing, China). Zhejiang Tianhang Bio (Zhejiang, China) supplied the fetal bovine serum. The CCK-8 kit was supplied by Glpbio (Shanghai, China).

### 3.2. Extraction and Purification of PFSP-2-1

After crushing, the *Perilla* seeds were sieved through 60-mesh screens. They were defatted with petroleum ether (1:20, *m*/*v*) for 6 h, followed by extraction with distilled water (1:20, *m*/*v*) at 85 °C for 2.5 h. The extraction was repeated three times, and then the extracts of the polysaccharides were combined. After evaporation and concentration under reduced pressure, anhydrous ethanol at the final concentration of 75% was added for alcohol precipitation, and the precipitate was centrifuged overnight at 4 °C and frozen, obtaining the crude polysaccharide of the *Perilla* seeds. After dissolving the crude polysaccharide in deionized water, free protein was precipitated using the Sevage method, and the supernatant was collected and freeze-dried. Decolorization was performed using the polyamide chromatography column method [42], and the eluate was collected, concentrated through evaporation under reduced pressure, and freeze-dried, yielding the *Perilla* seed polysaccharide (PFSP).

After dissolving 50 mg of PFSP in 5 mL of water, the solution was added to an activated equilibrated DEAE-52 cellulose column, which was sequentially eluted with a NaCl gradient solution (0–0.8 M). An eluate of the *Perilla* seeds was collected, concentrated, dialyzed, and lyophilized to obtain a polysaccharide fraction. According to previously obtained experimental results revealing the antitumor activity of the *Perilla* seed polysaccharide, Sephacryl S-500 HR column chromatography was selected for the further purification of PFSP-2. In this study, we selected the fraction PFSP-2-1 for structural characterization and verified its antitumor activity using the CCK-8 cell viability assay.

### 3.3. Structural Characterization of PFSP-2-1

#### 3.3.1. Physicochemical Characterization of PFSP-2-1

The phenol–sulfuric acid method was used to determine the polysaccharide content of PFSP-2-1, using d-glucose as a standard. Using d-glucuronic acid as the standard, uronic acid was detected via a carbazole reaction [43].

#### 3.3.2. Relative Molecular Weight (Mw) of PFSP-2-1

Dextran strands with different relative molecular weights and the polysaccharide PFSP-2-1 were weighed precisely, dissolved to obtain 5 mg/mL solutions, and centrifuged for 10 min at 12,000× *g*. The supernatant was filtered and transferred to the injection vial. The purity and the molecular weight of PFSP-2-1 were analyzed using a GPC system equipped with a Shimadzu RID-10A differential refractive index detector and connected to a BRT 105-104-102 tandem gel column (8 × 300 mm). The column temperature was maintained at 40 °C, the flow rate was maintained at 0.6 mL/min, and the injection volume was 20 µL [44].

#### 3.3.3. Monosaccharide Composition Analysis

To detect the monosaccharide composition of PFSP-2-1, an ICS5000 ion chromatograph (Billerica, MA, USA) equipped with a Dionex Carbopac TMPA 20 column (3 × 150) was used. Then, 10.0 mg of the polysaccharide PFSP-2-1 and 16 monosaccharide standards (rhamnose, fucose, galactose, arabinose, xylose, glucose, mannose, ribose, fructose, glucuronic acid, galacturonic acid, amino galactose hydrochloride, *N*-acetyl-glucosamine, glucosamine hydrochloride, mannuronic acid, and gulo-glucuronic acid) were precisely weighed in ampoules, before adding 10 mL of 3 M TFA. The ampoules were sealed under nitrogen purge, and the samples were hydrolyzed at 120 °C for 3 h. The acid hydrolysis solution was transferred to a glass tube through aspiration, and 10 mL of deionized water was added before mixing; then, 100 µL of the solution was aspirated, and 900 µL of deionized water was added. The supernatant was centrifuged at 12,000× *g* for 5 min, and the monosaccharide composition of PFSP-2-1 was determined using an ion chromatograph equipped with a Dionex Carbopac TMPA20 (3 × 150) column [45].

#### 3.3.4. Methylation Analysis

The methylation analysis of PFSP-2-1 was performed according to the method of Xie et al. [46]. Firstly, 2.0–3.0 mg of the polysaccharide PFSP-2-1 was added to a glass reaction flask, along with 1 mL of anhydrous DMSO, before quickly adding the methylation reagent A solution (anhydrous base solution). The flask was closed, the sample was dissolved using ultrasonic waves, and the methylation reagent B solution (iodomethane solution) was added. The mixture was stirred in a water bath at 30 °C for 60 min, before 2 mL of ultrapure water was added to stop the methylation reaction. At this point, 1 mL of 2 M trifluoroacetic acid (TFA) was added to the methylated polysaccharide to hydrolyze it, letting the reaction proceed for 90 min, and then the sample was concentrated through rotary evaporation. The residue was reduced by adding 2 mL of double-distilled water and 60 mg of sodium borohydride, and it was left to react for 8 h. Glacial acetic acid was added to neutralize the residue; the sample was evaporated, concentrated, and dried in a drying oven at 101 °C, and 1 mL of acetic anhydride was added to react at 100 °C for 1 h. The sample was then cooled. After adding 3 mL of toluene, the sample was concentrated under reduced pressure, and the excess acetic anhydride was removed through evaporation. The procedure was repeated 4–5 times. The acetylated product was dissolved with chloroform to obtain derivatives, which were analyzed using GCMS-QP 2010 gas chromatography/mass spectrometry (Kyoto, Japan) with an RXI-5 SIL MS column (30 m × 0.25 mm × 0.25 µm).

#### 3.3.5. FT-IR and NMR Spectroscopy

The dried PFSP-2-1 (1.0 mg) was combined with KBr to obtain clear, pressed tablets, which were then scanned in the range of 4000–400 cm^−1^ using FT-IR (Waltham, MA, USA) to analyze the organic functional groups of PFSP-2-1 [47].

PFSP-2-1 (50 mg) was weighed, dissolved in 0.5 mL of D_2_O, and freeze-dried, and we repeated the procedure twice. The samples were then dissolved in 0.5 mL of D_2_O, and ^1^H-NMR, ^13^C-NMR, chemical shift correlation (COSY), heteronuclear single quantum coherence (HSQC), and two-dimensional NOE spectra (NOESY) were obtained at 25 °C using a Bruker 600 NMR instrument (Billerica, MA, USA).

#### 3.3.6. Microscopic Analysis

A suitable quantity of polysaccharide powder was dissolved in ultrapure water. The sample was then diluted and dispersed well, dropped onto a smooth mica sheet, dried at room temperature, and tested [48]. Scanning was performed using atomic-pressure microscopy (Moscow, Russia); the probe was mounted, the laser was adjusted, and the sample stage and probe were moved such that the sample was located directly under the probe until the surface of the sample was visible. The Nanoscope software was used for the analysis.

#### 3.3.7. Triple-Helix Structure Determination

The triple-helix structure of PFSP-2-1 was determined using Congo red. Firstly, 5 mg of PFSP-2-1 was dissolved in 2 mL of a Congo red (80 μmol/L) solution. Six experimental groups were set up, to which 1 mol/L of NaOH was added, obtaining final NaOH concentrations of 0, 0.1, 0.2, 0.3, 0.4, and 0.5 mol/L. After mixing well, the control group was left to stand for 10 min. The control group was treated as the experimental group, with the exception that PFSP-2-1 was not added. A full-wavelength scan was performed in the 300–700 nm range, using a UV–visible spectrophotometer (Waltham, MA, USA) [49].

### 3.4. Evaluation of Antitumor Activity

#### 3.4.1. Cell Culture

HepG2 cells were cultured in high-sugar DMEM containing 10% fetal bovine serum and 1% double antibody. Hep3b cells were cultured in MEM containing 10% fetal bovine serum and 1% double antibody. SK-Hep-1 cells were cultured in RPMI-1640 medium containing 10% fetal bovine serum and 1% double antibody. The cell cultures were maintained at 37 °C in a 5% CO_2_ atmosphere.

#### 3.4.2. Cytotoxicity Experiment

The toxicity of PFSP-2-1 and PFSP-2-2 in the three tumor cell lines (HepG2, Hep3b, and SK-Hep-1) was assessed using a CCK-8 assay. In brief, the tumor cells were seeded in a 96-well plate at a density of 1 × 10^4^ cells/mL. The medium without polysaccharides was used as the control group. The polysaccharide samples PFSP-2-1 and PFSP-2-2 at five concentrations (100, 200, 400, 800, and 1600 µg/mL) were added to the cells on the 96-well plate. Using the CCK-8 kit [50], cell viability was calculated by measuring each well’s absorbance (OD) at 450 nm.

### 3.5. Statistical Analysis

SPSS 27.0 software was used to analyze the experimental data. In this paper, the results are expressed as the mean ± standard deviation, and the difference between different treatments was compared via one-way ANOVA, and multiple comparisons between each treatment were performed using the Tukey test method at *p* < 0.05.

## 4. Conclusions

PFSP-2-1 was isolated from *Perilla* meal as an effective antitumor polysaccharide fraction. The composition and structure of PFSP-2-1 were analyzed through FT-IR, GC–MS, IC, NMR, and AFM, and its antitumor activity was determined. The results showed that PFSP-2-1 is an acidic polysaccharide with a glyoxylate content of 16.22% ± 0.15% and a relative molecular weight of 8.81 × 10^6^ Da. Its composition was found to consist of arabinose, galactose, glucose, xylose, and glucuronide. Its main chain structure was →1)-Araf-(5→1)-Galp-(6→1)-Galp-(6→1)-Araf-(5→1)-Araf-(5→1)-Galp-(6→1)-Araf-(5→1)-Araf-(5→1)-Araf-(3→1)-Xylp-(4→, and its side-chain structures were →1,6)-Galp-(3→1)-Arap, →1,6)-Galp-(3→1,3)-Galp-(6→1)-Araf, →1,6)-Galp-(3→1)-Arap, and →1,3)-Araf-(5→1)-Glcp-(4→1)-Galp-(3→1)-Glcp, suggesting a three-dimensional helical structure. In vitro, we observed that PFSP-2-1 exerted proliferation-inhibitory effects on HepG2, Hep3b, and SK-Hep-1 cells in a dose-dependent manner. Thus, the results of our study lay a theoretical foundation for the elucidation of the molecular mechanism of the antitumor activity of *Perilla* seed polysaccharides and provide theoretical support for the development of *Perilla* seed polysaccharides as antitumor drugs.

## 5. Limitation

The limitations of this experiment are as follows:

(1) We did not compare the structure of polysaccharides via different extraction methods and, thus, could not possibly understand the entire structure of polysaccharides;
(2) This study only investigated the antitumor activity of polysaccharides from *Perilla* frutescens, but the mechanism of antitumor activity will need to be explored in the further.

## Figures and Tables

**Table 1:** Results of PFSP-2-1 methylated sugar alcohol acetyl ester (PMAA) analysis.

Retention Time (min)	Methylated Sugar	Mass Fragments (*m*/*z*)	Molar Ratio	Linage Pattern
17.040	2,3,5-Me3-Araf	43, 71, 87, 101, 117, 129, 145, 161	0.054	Araf-(1→
18.679	2,3,5-Me3-Arap	43, 71, 87, 101, 117, 129, 145, 161	0.238	Arap-(1→
22.895	2,3,5-Me3-Arap	43, 71, 87, 99, 101, 117, 129, 161, 189	0.503	→5)-Araf-(1→
23.081	2,3-Me2-Xylp	43, 71, 87, 99, 101, 117, 129, 161, 189	0.012	→4)-Xylp-(1→
25.036	2,3,4,6-Me4-Glcp	43, 71, 87, 101, 117, 129, 145, 161, 205	0.007	Glcp-(1→
26.967	2-Me1-Araf	43, 58, 85, 99, 117, 127, 159, 201	0.008	→3,5)-Araf-(1→
31.079	2,3,6-Me3-Glcp	43, 87, 99, 101, 113, 117, 129, 131, 161, 173, 233	0.005	→4)-Glcp-(1→
31.639	2,4,6-Me3-Galp	43, 87, 99, 101, 117, 129, 161, 173, 233	0.037	→3)-Galp-(1→
34.249	2,3,4-Me3-Galp	43, 87, 99, 101, 117, 129, 161, 189, 233	0.003	→6)-Galp-(1→
40.762	2,4-Me2-Galp	43, 87, 117, 129, 159, 189, 233	0.132	→3,6)-Galp-(1→

**Table 2:** ^1^H-NMR and ^13^C-NMR spectral assignments for PFSP-2-1 (ppm).

	Sugar Residues	H1/C1	H2/C2	H3/C3	H4/C4	H5/C5	H6/C6
A	Araf-(1→	5.09/108.73	4.45/103.22	3.91/66.87	3.98/83.77	3.62/61.02	3.54
B	Arap-(1→	4.45/105.42	—	—	—	—	—
C	→5)-Araf-(1→	5.09/106.95	4.04/81.17	3.94/76.62	4.07/86.59	3.83/67.09	—
D	→4)-Xylp-(1→	5.09/106.95	3.36/73.15	3.54/75.54	3.83/67.09	4.13/54.31	—
E	Glcp-(1→	4.35/103.22	3.98/83.77	4.09/82.47	3.54/65.36	3.80/68.60	4.08
F	→3,5)-Araf-(1→	5.10/106.78	4.07/86.59	4.04/81.17	3.80/68.60	3.54/65.36	—
G	→4)-Glcp-(1→	4.44/104.94	3.23/65.14	3.62/61.02	3.80/68.60	3.91/66.87	—
H	→3)-Galp-(1→	4.46/104.99	3.62/61.02	3.98/83.77	4.05/87.24	4.07/86.59	3.83
I	→6)-Galp-(1→	4.46/104.63	3.62/61.02	3.80/68.60	3.94/76.62	3.89/65.14	4.09
J	→3,6)-Galp-(1	4.45/103.22	3.92/68.60	3.63/61.03	4.01/81.17	3.85/67.09	3.85

**Table 3:** Difference analysis of cell survival rates of two polysaccharides at different concentrations.

Cell	Concentration (μg/mL)							F	*p*
	Polysaccharide	Control	100	200	400	800	1600		
HepG2	PFSP-2-1	104.99 ± 5.84 a	96.12 ± 2.05 ab	94.73 ± 1.36 b	87.98 ± 2.54 c	71.86 ± 3.12 d	53.34 ± 6.62 e	64.78808	<0.001
	PFSP-2-2	104.99 ± 5.84 a	100.37 ± 6.95 ab	94.73 ± 3.21 bc	90.34 ± 4.50 c	80.62 ± 4.08 d	61.07 ± 3.89 e	31.55905	<0.001
Hep3b	PFSP-2-1	100.00 ± 5.29 a	98.41 ± 3.59 a	95.04 ± 4.00 ab	89.74 ± 4.26 b	78.16 ± 6.40 c	70.33 ± 1.76 c	21.52656	<0.001
	PFSP-2-2	100.00 ± 5.29 a	97.32 ± 2.03 ab	94.74 ± 3.82 ab	90.52 ± 4.46 bc	85.01 ± 5.85 c	75.58 ± 4.92 d	11.73893	<0.001
SK-Hep-1	PFSP-2-1	100.00 ± 2.00 a	97.77 ± 1.37 ab	94.01 ± 2.82 b	88.62 ± 2.51 c	84.31 ± 3.39 c	71.06 ± 3.82 d	44.13431	<0.001
	PFSP-2-2	100.00 ± 2.00 a	97.26 ± 3.75 a	93.11 ± 4.20 a	92.73 ± 6.48 a	73.94 ± 3.96 b	64.02 ± 4.89 c	32.00001	<0.001

Note: Data in the table are presented as mean ± standard error. Different lowercase letters indicate the inhibitory effects of polysaccharides on tumor cells at different concentrations.

## Data Availability

Not applicable.
